# Supplementary material for: Tuberculosis and Post-Tuberculosis Lung Changes Are Associated with Exacerbations and Mortality in Chronic Obstructive Pulmonary Disease: A Population-Based Retrospective Cohort Study
Source: J Pers Med. 2026 Jun 29;16(7):351. doi: 10.3390/jpm16070351 (PMC13412321; doi:10.3390/jpm16070351)
Supplement: Supplementary file 1 [file jpm-16-00351-s001.zip › jpm-4314599.pdf]

Journal of
Personalized Medicine

Article

Tuberculosis and Post-Tuberculosis Lung Changes Are Associated with Exacerbations and Mortality in Chronic Obstructive Pulmonary Disease: A Population-Based Retrospective Cohort Study

Dmitry Oskin [1] and Stanislav Kotlyarov [2,*]

[1] Department of Infectious Diseases and Phthisiology, Ryazan State Medical University, 390026 Ryazan, Russia; doctor.oskin@yandex.ru
[2] Department of Nursing, Ryazan State Medical University, 390026 Ryazan, Russia; skmr1@yandex.ru
* Correspondence: skmr1@yandex.ru

Abstract

Background: Chronic obstructive pulmonary disease (COPD) and tuberculosis (TB) are among the most prevalent respiratory disorders worldwide and frequently coexist in the same patient. However, the contribution of active TB and post-tuberculosis lung disease to COPD exacerbations and long-term prognosis remains incompletely defined. **Objective**: To evaluate the prevalence, clinical correlates, and prognostic significance of tuberculosis and its sequelae in patients with COPD. **Materials and methods**: We conducted a population-based retrospective cohort study using de-identified data from the regional healthcare information system. The cohort included all adults aged 18 years or older with a recorded diagnosis of COPD (ICD-10 code J44). Tuberculosis was identified by codes A15–A19 and B90. The primary outcomes were COPD exacerbations and all-cause mortality. Group comparisons, cluster analysis, Kaplan–Meier survival analysis, Cox proportional hazards modeling, and multivariable logistic regression were performed. **Results**: Tuberculosis and/or its sequelae were identified in 267 of 16,714 patients (1.60%): post-TB sequelae (B90) in 197 (73.8%), active TB (A15–A19) in 22 (8.2%), and both in 48 (18.0%). Compared with patients without TB, those with COPD-TB were younger (63.5 ± 14.2 vs. 65.7 ± 14.7 years; $p = 0.018$), more often male (75.3% vs. 52.0%; $p < 0.001$), and had higher mortality (16.5% vs. 10.6%; $p = 0.003$). COPD-TB was associated with bronchiectasis (OR = 6.07; 95% CI, 3.03–12.16), pulmonary fibrosis (OR = 5.67; 95% CI, 3.40–9.45), and pneumonia (OR = 2.01; 95% CI, 1.50–2.71), but with lower prevalences of obesity, diabetes mellitus, and hypertension. Patients with TB experienced more COPD exacerbations, including recurrent exacerbations. In multivariable models, tuberculosis was associated with COPD exacerbations after adjustment for age and sex (adjusted OR = 1.43; 95% CI, 1.05–1.96); this association was attenuated and lost significance after further adjustment for post-tuberculosis structural lung disease, indicating that it is largely mediated by post-TB sequelae. Tuberculosis remained associated with mortality after adjustment for available covariates, both in logistic regression (adjusted OR = 1.61; 95% CI, 1.14–2.28) and in Cox analysis (hazard ratio = 1.37; 95% CI, 1.01–1.85). **Conclusions**: Tuberculosis and post-tuberculosis lung disease are clinically accessible risk markers associated with COPD exacerbations and mortality. These findings support recognizing patients with COPD and a history of TB as a high-risk subgroup requiring intensified follow-up, proactive exacerbation prevention, and prioritized vaccination counseling. In the context of personalized

Academic Editor(s): Marijn Speeckaert

Received: 27 April 2026
Revised: 10 June 2026
Accepted: 28 June 2026
Published: date

Copyright: © 2026 by the authors. Submitted for possible open access publication under the terms and conditions of the Creative Commons Attribution (CC BY) license.

medicine, a documented history of tuberculosis and post-tuberculosis lung changes represents a clinically accessible marker that can be used to stratify individual risk and to tailor monitoring and prevention in patients with COPD.

Keywords: chronic obstructive pulmonary disease; tuberculosis; post-tuberculosis lung disease; comorbidity; COPD exacerbations; mortality; Cox regression; population-based cohort study; COPD phenotyping; Voronezh Region; personalized medicine; risk stratification; precision medicine

1. Introduction

Chronic obstructive pulmonary disease (COPD) is one of the leading chronic respiratory disorders worldwide. Its prevalence in the adult population is commonly estimated at around 10%, and it remains among the leading causes of death globally [1–4]. A hallmark of COPD is its marked clinical heterogeneity, with substantial variation in symptoms, exacerbation burden, comorbidities, and long-term outcomes. This heterogeneity has driven sustained interest in clinically meaningful phenotyping of the disease [5,6].

Over the past decade, pulmonary tuberculosis has increasingly been recognized as an important determinant of COPD phenotype and prognosis [7,8]. Tuberculosis remains a major global health problem: according to the World Health Organization, 10.6 million new cases and 1.3 million deaths were recorded worldwide in 2022 [9]. Beyond acute disease, prior TB may lead to persistent structural and functional lung impairment, now commonly referred to as post-tuberculosis lung disease (PTLD) [10,11]. PTLD includes fibrosis, bronchiectasis, residual cavitation, chest wall changes, and chronic airflow limitation-abnormalities that may aggravate COPD severity, increase exacerbation risk, and worsen long-term outcomes [12,13]. Epidemiological studies indicate that a history of tuberculosis and residual post-TB lung changes are encountered more frequently among patients with COPD than in the general population [14,15]. In some regions, 20–45% of patients with COPD have documented prior TB or CT findings consistent with previous tuberculous infection [14]. Meta-analyses have demonstrated that previous tuberculosis increases the risk of developing COPD approximately 2.5- to 3-fold compared with individuals without TB [16,17], while a recent systematic review including 32 studies and over 670,000 patients confirmed a bidirectional association, with COPD prevalence among TB survivors reaching 15–21% [18]. Moreover, COPD–TB comorbidity is associated with a more severe disease course: affected patients tend to be younger at disease onset, are hospitalized more frequently for acute exacerbations, and have worse long-term outcomes, including more rapid lung function decline and higher mortality [19,20]. The first severe COPD exacerbations in patients with tuberculosis-associated COPD have been shown to occur significantly earlier than in those without a TB history [8,21]. Taken together, these observations suggest that the coexistence of COPD and tuberculosis constitutes a distinct clinicopathogenetic disease variant in which post-infectious lung damage contributes substantially to symptom burden and disease progression [22,23]. This comorbidity is associated with increased risks of adverse outcomes—exacerbations, respiratory failure, and death—and warrants particular attention during follow-up, including active TB case-finding and close monitoring of pulmonary function to guide timely therapeutic adjustment [12]. Despite this growing evidence, population-based data on the prognostic impact of TB and post-TB sequelae in COPD remain limited, particularly in regions with intermediate TB burden. The aim of the present study was to evaluate the prevalence, clinical correlates, and prognostic significance of tuberculosis and its sequelae in patients with COPD using regional population-based registry data. Because a history of tuberculosis and post-

tuberculosis lung changes are routinely documented and clinically accessible characteristics, they are attractive candidates for individual-level risk stratification in COPD. Conceptualizing them as personal, identifiable markers—rather than as generic comorbidities—is directly aligned with the goals of personalized respiratory medicine, in which the intensity of follow-up, monitoring, and preventive measures is matched to each patient's individual risk profile.

2. Materials and Methods

This population-based retrospective cohort study analyzed healthcare utilization data for patients with COPD treated in the Voronezh Region between 2019 and 2025. The study was approved by the Local Ethics Committee of Ryazan State Medical University, Russian Ministry of Health (Protocol No. 4, 6 December 2024). The Ethics Committee approved the use of data from 2019 to 2025 obtained from the regional health information system. The source dataset comprised all patients recorded in the regional health information system with ICD-10 code J44. The final cohort included 16,714 adults aged 18 years or older. Tuberculosis was identified using ICD-10 codes A15–A19 for active disease and B90 for post-tuberculosis sequelae. Patients were classified as having COPD-TB if either current or prior TB was documented at any point during the observation period. Cases of active tuberculosis (A15–A19) and post-tuberculosis sequelae (B90) were combined into a single analytical category ("tuberculosis and/or its sequelae"). This approach was adopted because both active tuberculous disease and residual structural lung damage—including bronchiectasis, fibrosis, and bronchial distortion—affect the course of COPD through shared pathogenetic mechanisms: impaired mucociliary clearance, microbial persistence in structurally altered airways, and sustained chronic inflammation. This rationale is consistent with the concept of tuberculosis-associated COPD (TB-COPD), which considers the full spectrum of tuberculous lung involvement as a unifying etiological factor for airflow obstruction, and aligns with the GOLD 2025 etiotype classification, in which the infectious/post-tuberculosis variant is designated as COPD-I [24]. In addition, the boundary between "active" and "cured" tuberculosis in ICD-10 coding is often ambiguous in routine clinical practice: some patients carry codes from both groups simultaneously because of recurrent disease or coding conventions. The small number of patients with isolated active tuberculosis (n = 22) also precluded a separate subgroup analysis with adequate statistical power. A descriptive characterization of TB structure (active, sequelae, or both), including demographic and mortality data for each subgroup, is provided in the Results section.

The source dataset was obtained from the regional health information system, which records all healthcare encounters across all healthcare organizations in the region. Data are entered by treating physicians, with all diagnoses established in accordance with current clinical guidelines; physicians are trained to use the system, and the quality of data entry is monitored within each institution [25]. By study design, only de-identified data on the dates of medical encounters and the corresponding ICD-10-coded diagnoses were available to the investigators; other clinical data, including examination and laboratory results, were not included in the present analysis. The dataset was fully de-identified by the authorized data custodian before transfer, and the research team analyzed only these anonymized records, with no access to names, identifiers, or any key enabling re-identification. Beyond quantifying the overall prognostic impact of tuberculosis, these population-based data were used to delineate clinically distinguishable subgroups of patients with COPD—through comorbidity-based clustering and stratified analyses—that differ in their expected burden of exacerbations and mortality, in line with a personalized-medicine approach in which monitoring and prevention are matched to an individual patient's risk profile.

https://doi.org/10.3390/xxxxx

The primary study outcomes were COPD exacerbations and all-cause mortality during follow-up. Exacerbations were identified using ICD-10 codes J44.0 and J44.1. We assessed the proportion of patients with at least one exacerbation, the mean number of COPD exacerbations per patient, and the distribution of exacerbation frequency categories. All-cause mortality was analyzed with both cumulative event analyses and time-to-event methods. COPD exacerbations were ascertained from all dated J44.0 and J44.1 records in the regional health information system, capturing both outpatient and inpatient encounters; each such record was counted as a separate exacerbation episode, and frequent COPD exacerbations were defined as at least two registered episodes during follow-up. Because the registry does not reliably distinguish exacerbation severity or care setting, COPD exacerbations were analyzed as coded events rather than as severity-graded events.

Continuous variables are presented as mean ± standard deviation and categorical variables as *n* (%). Categorical data were compared using the chi-square test. For continuous variables, group comparisons were performed according to distributional characteristics; in particular, the number of COPD exacerbations was compared using the Mann–Whitney U test because of non-normality. Odds ratios (ORs) with 95% confidence intervals (CIs) were calculated for binary outcomes. Survival was analyzed using Kaplan–Meier curves and Cox proportional hazards regression. To identify factors associated with mortality and COPD exacerbations after adjustment, multivariable logistic regression models were fitted with adjustment for age, sex, and major comorbid conditions. All analyses were performed in R version 4.6.0 (R Foundation for Statistical Computing, Vienna, Austria). Survival analyses (Kaplan–Meier and Cox proportional hazards models) were conducted with the survival and survminer packages; incidence rates with exact Poisson confidence intervals were calculated with epitools; baseline comparisons and standardized mean differences were computed with tableone; and K-means clustering and logistic regression were performed using the base-R functions kmeans and glm.

To characterize clinical heterogeneity within the cohort, we performed K-means clustering based on comorbidity profiles. The binary feature matrix included 24 comorbid conditions derived from ICD-10 codes and covering cardiovascular, metabolic, respiratory, oncologic, gastrointestinal, and infectious diseases. The number of clusters was selected on the basis of clinical interpretability and internal validity metrics. Solutions for k ranging from 2 to 8 were evaluated iteratively; at k ≤ 3, the profiles merged clinically heterogeneous groups, whereas at k ≥ 6, internally homogeneous clusters fragmented without yielding new clinically meaningful patterns. A five-cluster solution (k = 5) provided the optimal balance between clinical interpretability and within-cluster homogeneity. To improve stability, 25 random centroid initializations were used (nstart = 25), retaining the solution that minimized the total within-cluster sum of squares (iter.max = 100). Clustering was performed using the kmeans function in R (base package). Because k-means relies on Euclidean distance, which is not ideal for binary comorbidity indicators, the clustering was treated as an exploratory, descriptive step rather than a definitive partition; internal validity was modest across all solutions (mean silhouette width 0.13–0.16 for k = 2–8 in a 2000-patient random sample), and the five-cluster solution was retained primarily for clinical interpretability rather than on the basis of an optimal validity metric. Clusters were then compared with respect to age, sex, multimorbidity burden, and the distribution of tuberculosis (Figure 1).

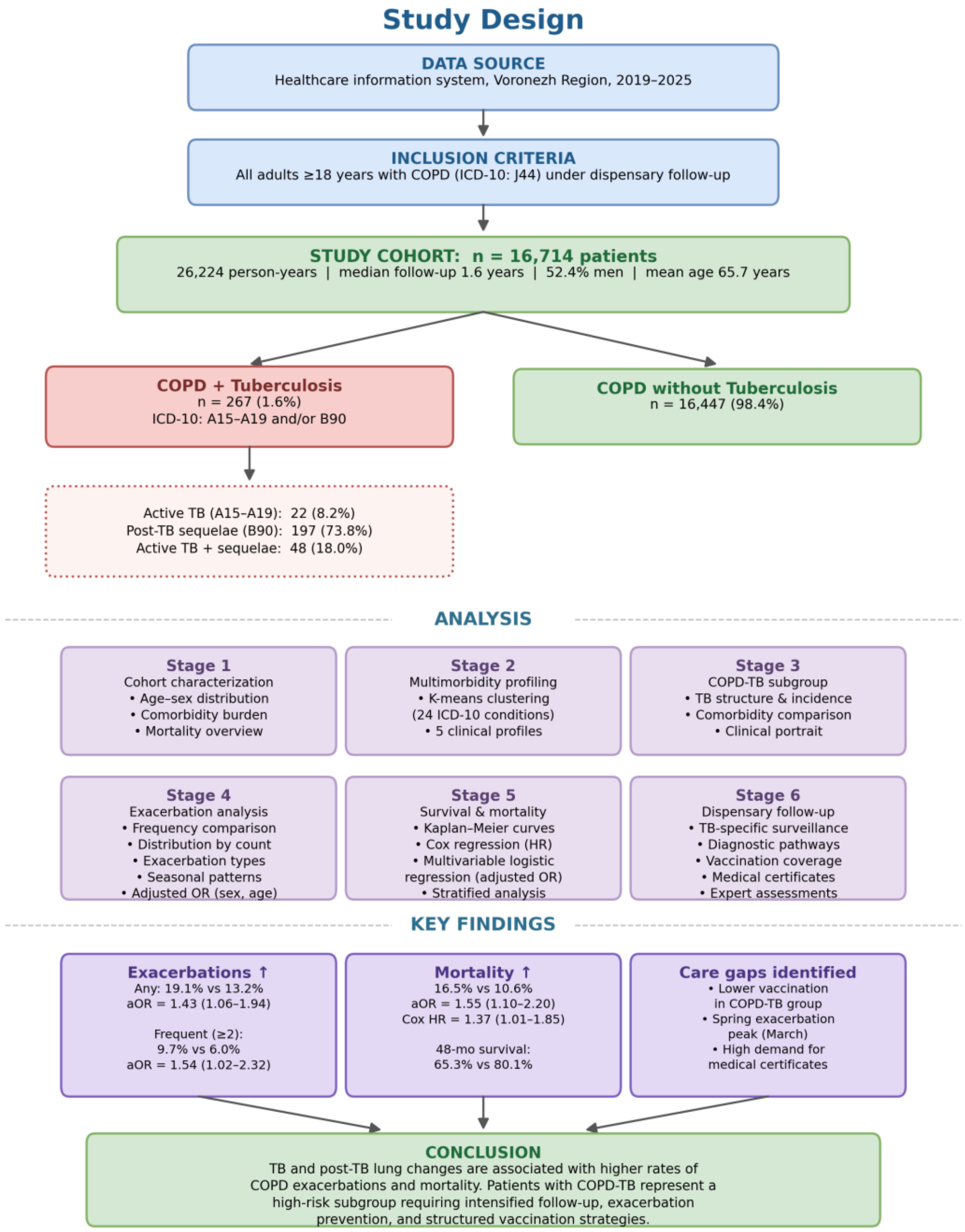

Figure 1. Study design. COPD, chronic obstructive pulmonary disease; TB, tuberculosis; ICD-10, International Classification of Diseases, 10th Revision. Box colours indicate the role of each element: blue, data source and eligibility criteria; green, the overall study cohort and the tuberculosis-free comparison group; red, the COPD-TB group of interest and its diagnostic composition; lilac, the analytical stages; violet, the principal findings. Colours are used for visual organisation only and convey no quantitative information.

3. Results.

3.1. General Characteristics of the COPD Cohort

The study included 16,714 patients with chronic obstructive pulmonary disease (ICD-10 code J44) who sought medical care in the Voronezh Region between 2019 and 2025.

Among the included patients, 8757 (52.4%) were men and 7957 (47.6%) were women. The mean age was 65.7 ± 14.7 years (median, 68.0 years; range, 18–104 years). The age and sex distribution of the cohort is shown in Supplementary Figure S1.

More than two-thirds of patients (74.8%) were aged 60 years or older, which is consistent with the typical age structure of COPD populations. The age–sex pattern was also characteristic: men predominated in age groups below 70 years (55–56%), the sex ratio approached parity in the 70–79-year group (51.6% men), and women predominated among patients aged 80 years or older (59.3%). This pattern may reflect both the longer life expectancy of women and the more aggressive course of COPD in men, associated with higher rates of smoking and occupational exposure.

Comorbidity analysis demonstrated a high burden of multimorbidity in the cohort (Supplementary Table S1). Cardiovascular disease was most prevalent: arterial hypertension was present in 80.5% of patients, cerebrovascular disease in 44.4%, and ischemic heart disease in 38.2%. Chronic heart failure was recorded in 8.0% and atrial fibrillation in 8.3%. Among infectious conditions, prior COVID-19 was common (29.5%), reflecting the impact of the pandemic on this population. Tuberculosis and its sequelae were recorded in 267 patients (1.6%), equivalent to approximately one in every 63 COPD patients. Diabetes mellitus was present in 14.8%, obesity in 11.4%, and malignant neoplasms in 13.1%, underscoring the need for oncologic vigilance in COPD. Asthma–COPD overlap was identified in 16.0% and chronic bronchitis in 21.2%.

During follow-up, 1793 patients died, corresponding to an overall all-cause mortality of 10.7% for the entire observation period (2019–2025).

3.2. Multimorbidity Profiles of Patients with COPD

To identify clinically meaningful subgroups with distinct patterns of multimorbidity, we performed K-means clustering. A five-cluster solution provided the best balance between clinical interpretability and within-cluster homogeneity; larger solutions produced only limited additional separation while substantially reducing practical interpretability (Supplementary Table S2).

The largest cluster was the cardiovascular profile ($n = 4402$; 26.3%), composed of older patients with marked cardiovascular pathology. Mean age was 71.8 years, the proportion of men was 54.8%, and the mean number of comorbidities was 4.2. The defining conditions were ischemic heart disease (100%), arterial hypertension (97%), cerebrovascular disease (61%), and malignant neoplasms (22%). This profile had the highest mortality of all clusters (14.0%), highlighting the adverse prognostic role of combined COPD and ischemic heart disease. TB prevalence was 1.4%, close to the cohort average (1.6%).

The cerebrovascular profile ($n = 2626$; 15.7%) was characterized by predominant cerebrovascular pathology with a relatively low ischemic heart disease prevalence. Mean age was 69.9 years; women accounted for 52.2%. The mean number of comorbidities was 3.0. Key conditions were cerebrovascular disease (100%), arterial hypertension (89%), diabetes mellitus (16%), and malignant neoplasms (16%). Mortality was 12.6%, and TB prevalence was 1.9%.

The hypertensive profile ($n = 4282$; 25.6%) consisted of comparatively younger patients (mean age, 64.1 years) with isolated arterial hypertension and a low burden of comorbidity (mean, 2.1 conditions). Sex distribution was close to parity (53.1% men). The main comorbidities were arterial hypertension (100%), cerebrovascular disease (17%), bronchial asthma (15%), and diabetes mellitus (15%). This profile had relatively low mortality (7.4%), suggesting a more favorable prognosis in the setting of controlled hypertension without severe cardiac disease. TB prevalence was 1.4%.

The polymorbid profile ($n = 2607$; 15.6%) had the highest number of comorbidities (mean, 5.4) and a marked predominance of women (60.1%). Mean age was 69.5 years. Key comorbidities included gastritis/peptic ulcer disease (100%), arterial hypertension (98%), cerebrovascular disease (80%), ischemic heart disease (66%), prior COVID-19 (32%), and atrial fibrillation (22%). Despite the greatest overall burden of comorbidity, this profile

https://doi.org/10.3390/xxxxx

had the lowest mortality (6.7%), suggesting a possible "multimorbidity paradox," potentially explained by closer follow-up and more regular pharmacotherapy in patients with multiple chronic diseases. TB prevalence was 1.5%.

The isolated COPD profile (*n* = 2797; 16.7%) comprised the youngest patients (mean age, 50.7 years), with the lowest comorbidity burden (mean, 0.8 conditions) and the highest proportion of men (63.5%). The most common associated conditions were prior COVID-19 (17%) and asthma (13%). Despite younger age and fewer comorbidities, mortality remained high (12.6%), probably reflecting a more severe intrinsic COPD course. Importantly, this profile showed the highest TB prevalence (2.1%), 1.3 times higher than the cohort average.

Thus, tuberculosis was most strongly linked to the isolated COPD profile, which consisted mainly of younger men without marked cardiovascular comorbidity. This observation has practical implications: patients with apparently "pure" COPD who lack the usual cardiometabolic comorbidity spectrum may nevertheless represent a clinically vulnerable subgroup if they have a history of TB. From a personalized-medicine standpoint, this means that a single, routinely recorded variable—a documented history of tuberculosis—can reclassify an otherwise low-comorbidity patient into a higher-risk COPD subgroup, supporting individualized rather than uniform follow-up.

3.3. Characteristics of the COPD + Tuberculosis Subgroup

Of the 16,714 patients with COPD, 267 had tuberculosis or its sequelae, corresponding to 1.6% of the total cohort. In other words, every 63rd patient with COPD had a history of tuberculosis. The TB structure within the COPD cohort is shown in Table 1. In the majority of cases (73.8%), the registered condition was sequelae of prior tuberculosis (ICD-10 code B90), whereas active respiratory TB (A15–A19) without sequelae accounted for only 8.2%. In 18.0% of cases, codes for both active TB and post-TB sequelae were present, suggesting recurrent or complex disease trajectories.

Table 1. Structure of tuberculosis among patients with COPD (*n* = 267).

TB Manifestation	ICD-10 Codes	*n*	%	Age, Years (Mean ± SD)	Men, %	Mortality, %
Active tuberculosis	A15–A19	22	8.2	67.8 ± 15.3	77.3	13.6
Post-TB sequelae	B90	197	73.8	64.6 ± 14.5	74.1	19.3
Active TB + sequelae	A15–A19, B90	48	18.0	57.3 ± 10.8	79.2	6.2
Total		267	100.0	63.5 ± 14.2	75.3	16.5

Mortality varied substantially across TB manifestations. The highest mortality (19.3%) was observed in patients with post-TB sequelae, whereas those coded for both active TB and sequelae had the lowest mortality (6.2%). This apparent paradox may reflect closer specialist follow-up in patients with recently active disease, whereas the long-term consequences of post-TB structural lung damage may be underestimated in routine care.

To assess the risk of active TB in patients with COPD, incidence rates were calculated using registry data for 2020–2024.

During follow-up, 69 newly diagnosed cases of active respiratory tuberculosis were recorded among patients with COPD. The overall incidence rate was 1.16 per 1000 person-years (95% CI, 0.91–1.47) (Supplementary Figure S2). Compared with the average annual TB incidence in the Russian Federation during the same period (approximately 30 per 100,000 population per year, or 0.30 per 1000), assumed to approximate person-years under a stationary-population assumption, the incidence among patients with COPD was

roughly fourfold higher (incidence rate ratio ≈ 3.9), supporting the concept of COPD as a clinically relevant risk condition for TB.

Patients with COPD-TB comorbidity differed significantly from those without tuberculosis across all three key demographic and prognostic parameters. They were younger (mean age 63.5 ± 14.2 vs. 65.7 ± 14.7 years; $p = 0.018$), were predominantly male (75.3% vs. 52.0%; $p < 0.001$), and had higher all-cause mortality (16.5% vs. 10.6%; $p = 0.003$).

The age–sex distribution of patients with COPD and tuberculosis is presented in Supplementary Figure S1.

In age groups below 70 years, the proportion of men among patients with COPD and TB reached 77–85%, markedly exceeding the corresponding values in the overall COPD cohort (55–56%). The sex ratio approached parity only in older age groups (70 years and older).

A comparative analysis of comorbidity frequencies revealed distinctive features of the comorbidity profile in patients with COPD and tuberculosis (Table 2).

Table 2. Baseline characteristics of patients with COPD according to tuberculosis status.

Characteristic	COPD-TB (*n* = 267)	COPD Without TB (*n* = 16,447)	*p*	SMD
Demographics and outcomes				
Age, years (mean ± SD)	63.5 ± 14.2	65.7 ± 14.7	0.018	0.15
Male sex, *n* (%)	201 (75.3)	8556 (52.0)	<0.001	0.50
≥1 exacerbation, *n* (%)	51 (19.1)	2164 (13.2)	0.006	0.16
Frequent exacerbations (≥2), *n* (%)	26 (9.7)	982 (6.0)	0.015	0.14
All-cause mortality, *n* (%)	44 (16.5)	1749 (10.6)	0.003	0.17
Comorbidities, *n (%)*				
Arterial hypertension	187 (70.0)	13,274 (80.7)	<0.001	0.25
Cerebrovascular disease	121 (45.3)	7300 (44.4)	0.808	0.02
Ischemic heart disease	94 (35.2)	6297 (38.3)	0.335	0.06
Atrial fibrillation	20 (7.5)	1368 (8.3)	0.708	0.03
Chronic heart failure	27 (10.1)	1313 (8.0)	0.247	0.07
Atherosclerosis	30 (11.2)	1161 (7.1)	0.012	0.15
Diabetes mellitus	21 (7.9)	2458 (14.9)	0.002	0.22
Obesity	11 (4.1)	1897 (11.5)	<0.001	0.28
Chronic bronchitis	73 (27.3)	3468 (21.1)	0.016	0.15
Asthma	45 (16.9)	2624 (16.0)	0.754	0.02
Pneumonia	57 (21.3)	1953 (11.9)	<0.001	0.26
Pulmonary fibrosis	17 (6.4)	195 (1.2)	<0.001	0.27
Emphysema	4 (1.5)	128 (0.8)	0.332	0.07
Bronchiectasis	9 (3.4)	94 (0.6)	<0.001	0.20
Prior COVID-19	67 (25.1)	4867 (29.6)	0.126	0.10
Hepatitis/chronic liver disease	15 (5.6)	432 (2.6)	0.005	0.15
Malignant neoplasm	39 (14.6)	2147 (13.1)	0.512	0.05
Rheumatoid arthritis	8 (3.0)	171 (1.0)	0.005	0.14

Note. Continuous variables compared with the t-test, categorical variables with the chi-square test. SMD, standardized mean difference (|SMD| > 0.1 indicates a meaningful imbalance). Reference: COPD without tuberculosis.

Patients with COPD and tuberculosis were significantly more likely to have structural lung abnormalities: bronchiectasis was nearly six times more common and pulmonary fibrosis more than five times more common. Pneumonia was also more frequent, which may reflect both structural bronchopulmonary damage and heightened susceptibility to infection.

A particularly important finding was the lower prevalence of metabolic disorders in the COPD-TB group: obesity was 2.8-fold less frequent, diabetes mellitus was 1.9-fold less frequent, and arterial hypertension was also less common. Taken together, these differences define a distinctive comorbidity pattern that differs from the usual cardiometabolic phenotype of COPD.

The data also allow construction of a clinical portrait of the typical patient with COPD-TB comorbidity. In our cohort, such a patient was most often a man (75.3%) approximately 64 years of age, younger than the average patient with COPD. In nearly three-quarters of cases, the relevant condition was not active TB but post-TB sequelae. Relative to patients with COPD alone, these individuals more frequently had bronchiectasis, fibrosis, pneumonia, and exacerbations, but less frequently had obesity, diabetes, and hypertension.

3.4. Effect of Tuberculosis on COPD Course: Exacerbations

COPD exacerbations are a key determinant of disease progression, quality of life, and prognosis. We therefore compared the frequency and pattern of exacerbations according to the presence of tuberculosis. The results are presented in Table 3.

Table 3. COPD exacerbations according to tuberculosis status.

Parameter	COPD-TB (*n* = 267)	COPD without TB (*n* = 16,447)	*p*
A. Exacerbation frequency			
Patients with ≥1 exacerbation, *n* (%)	51 (19.1)	2164 (13.2)	0.006
Number of exacerbations (mean ± SD)	0.52 ± 1.72	0.29 ± 1.43	0.004 *
Frequent exacerbations (≥2), *n* (%)	26 (9.7)	979 (6.0)	0.015
B. Distribution by number of exacerbations, *n (%)*			
0	216 (80.9)	14,283 (86.8)	
1	25 (9.4)	1182 (7.2)	
2	11 (4.1)	454 (2.8)	
3	7 (2.6)	223 (1.4)	
4	0 (0.0)	125 (0.8)	
≥5	8 (3.0)	180 (1.1)	
C. Exacerbation type, *n (%)*			
With acute LRTI (J44.0)	11 (4.1)	568 (3.5)	0.673
Unspecified (J44.1)	45 (16.9)	1749 (10.6)	0.002

Note. * Mann–Whitney U test. LRTI, lower respiratory tract infection.

COPD exacerbations were significantly more frequent in the group with concomitant tuberculosis. At least one exacerbation during follow-up was recorded in 19.1% of patients with COPD-TB versus 13.2% of those without TB ($\chi^2 = 7.57$; $p = 0.006$). The unadjusted odds ratio for any exacerbation was 1.56 (95% CI, 1.14–2.12); after adjustment for sex and age, the adjusted odds ratio was 1.43 (95% CI, 1.05–1.96; $p = 0.023$), indicating a 43% higher

https://doi.org/10.3390/xxxxx

exacerbation risk in the presence of TB after adjustment for demographic factors. The mean number of exacerbations per patient was also higher in the COPD-TB group (0.52 ± 1.72 vs. 0.29 ± 1.43; Mann–Whitney U test, $p = 0.004$), corresponding to an approximately 1.8-fold increase. Frequent exacerbations (≥2 during follow-up) occurred in 9.7% of TB-exposed patients versus 6.0% of non-exposed patients ($p = 0.015$). The unadjusted odds ratio for frequent exacerbations was 1.70 (95% CI, 1.13–2.56); after adjustment for sex and age, the aOR was 1.54 (95% CI, 1.02–2.32; $p = 0.041$).

A notably larger proportion of patients with multiple COPD exacerbations (≥5 during follow-up) was observed in the COPD-TB group (3.0% vs. 1.1%), suggesting the emergence of a frequent exacerbator phenotype in a subset of patients with this comorbidity.

Statistically significant differences were found for exacerbations coded as J44.1 (“chronic obstructive pulmonary disease with unspecified COPD exacerbation”): 16.9% in the COPD-TB group versus 10.6% in the COPD-only group ($p = 0.002$). The frequency of COPD exacerbations associated with acute lower respiratory tract infection (J44.0) did not differ significantly (4.1% vs. 3.5%; $p = 0.673$). The predominance of “unspecified” exacerbations in patients with TB may reflect diagnostic complexity: COPD exacerbation symptoms in individuals with post-tuberculosis lung damage may mask or mimic infectious processes, making etiologic verification more difficult.

Taken together, these findings indicate that tuberculosis, including post-TB sequelae, is a clinically meaningful marker associated with COPD exacerbations. The higher exacerbation burden in patients with COPD-TB may be mediated by structural lung damage such as bronchiectasis, fibrosis, and bronchial distortion, along with chronic inflammation and impaired mucociliary clearance.

Because bronchiectasis was uncommon overall and present in only nine patients in the COPD-TB group, a formal analysis stratified by bronchiectasis status was statistically unstable; we therefore assessed the mediating role of structural and infectious sequelae by adjustment rather than stratification. To examine whether this excess exacerbation risk was explained by post-tuberculosis structural lung damage, we added bronchiectasis, pulmonary fibrosis, and pneumonia to the multivariable model. The association between tuberculosis and at least one exacerbation, significant after adjustment for age and sex alone (adjusted OR = 1.43; 95% CI, 1.05–1.96), was attenuated and no longer statistically significant after further adjustment for these structural and infectious conditions (adjusted OR = 1.27; 95% CI, 0.93–1.74; $p = 0.14$); a similar attenuation was observed for frequent exacerbations (from adjusted OR = 1.53 to 1.29; 95% CI, 0.85–1.97). This pattern indicates that the higher exacerbation burden in COPD-TB is largely mediated by post-tuberculosis structural sequelae (bronchiectasis and fibrosis) and a greater susceptibility to pneumonia, rather than reflecting an effect of tuberculosis independent of these sequelae. By contrast, the association of tuberculosis with mortality persisted after the same adjustment (adjusted HR = 1.44; 95% CI, 1.06–1.94). These findings are consistent with a mechanistic pathway in which prior tuberculosis predisposes to COPD exacerbations through chronic structural lung damage, reinforcing post-tuberculosis lung disease as the actionable target for exacerbation prevention in this subgroup.

To assess the role of seasonality in COPD course, exacerbations were analyzed by calendar month and season. A total of 4995 exacerbation episodes were evaluated: 138 in the COPD-TB group and 4857 in the COPD-only group. Month-by-month distribution is shown in Supplementary Figure S4.

Marked differences were observed in peak months. In the COPD-TB group, exacerbations peaked in March (15.2%), whereas in the COPD-only group, the peak occurred in November (9.6%). The proportion of March exacerbations was numerically higher among patients with TB history, suggesting a shift in the seasonal pattern of exacerbations in this subgroup.

https://doi.org/10.3390/xxxxx

Overall seasonal distribution did not differ significantly between groups (chi-square = 2.80; $p = 0.424$). However, patients with COPD-TB tended to experience a greater proportion of exacerbations in spring and a lower proportion in autumn. Although exploratory, this pattern suggests that TB-related structural and immunologic sequelae may modify seasonal vulnerability.

The spring shift in exacerbation peak among patients with COPD and tuberculosis may be related to several mechanisms. Spring has been linked to increased TB reactivation, potentially because of post-winter immune vulnerability, hypovitaminosis, and reduced solar exposure. Patients with post-TB lung disease may also have seasonally amplified susceptibility to respiratory infections and inflammatory destabilization.

These seasonal findings should be regarded as hypothesis-generating; they do not establish a causal seasonal mechanism. If confirmed in prospective studies, the spring shift might inform the timing of follow-up visits and preventive counseling (including influenza and pneumococcal vaccination) in patients with COPD-TB; however, no change in practice is warranted on the basis of these exploratory data.

3.5. Effect of Tuberculosis on Prognosis: Survival Analysis

During follow-up (2019–2025), death occurred in 44 of 267 patients with COPD and tuberculosis (16.5%) compared with 1749 of 16,447 patients without TB (10.6%). The difference was statistically significant ($\chi^2 = 8.77$; $p = 0.003$). The unadjusted odds ratio for death was 1.66 (95% CI, 1.20–2.30), while the sex- and age-adjusted odds ratio was 1.61 (95% CI, 1.14–2.28; $p = 0.007$). Cox regression performed in 15,921 patients with available follow-up time (total, 26,224 person-years; median follow-up, 1.6 years) confirmed that tuberculosis remained associated with mortality after adjustment: HR = 1.37 (95% CI, 1.01–1.85; $p = 0.040$), adjusted for sex (HR = 2.83) and age (HR = 1.057 per year). Kaplan–Meier curves showed a significant separation between groups (log-rank $p = 0.002$). Patients were followed from the first recorded J44 code to the date of the last recorded healthcare contact or death; those without an observable follow-up interval (no recorded contact after cohort entry) were excluded from the time-to-event analysis. The proportional-hazards assumption was satisfied for the effect of tuberculosis (Schoenfeld residuals, $p = 0.73$) and sex ($p = 0.44$); the global test was significant owing to a non-proportional effect of age ($p = 0.003$), which does not affect the interpretation of the tuberculosis hazard ratio. Cumulative survival estimates are shown in Figure 2.

Differences in survival widened over time. At 12 months, the absolute survival gap was 2.8 percentage points (91.3% vs. 94.1%), whereas by 48 months, it had increased to 14.8 percentage points (65.3% vs. 80.1%). This pattern suggests that TB history is associated not only with short-term vulnerability but also with cumulative long-term disadvantage.

The age-stratified mortality analysis revealed heterogeneity in the impact of TB on prognosis (Supplementary Table S5).

A statistically significant increase in mortality risk associated with TB was observed in the 70–79-year age group (OR = 2.34; 95% CI, 1.36–4.02; $p = 0.003$). In this stratum, mortality in patients with COPD-TB reached 25.7%, highlighting a particularly vulnerable subgroup for intensified follow-up.

Mortality among men with COPD-TB was 19.4%, which was 2.5 times higher than among women with the same comorbidity (7.6%). This difference is consistent with the broader pattern of higher COPD mortality in men and may reflect cumulative smoking exposure, occupational risk, and lower adherence to preventive care.

https://doi.org/10.3390/xxxxx

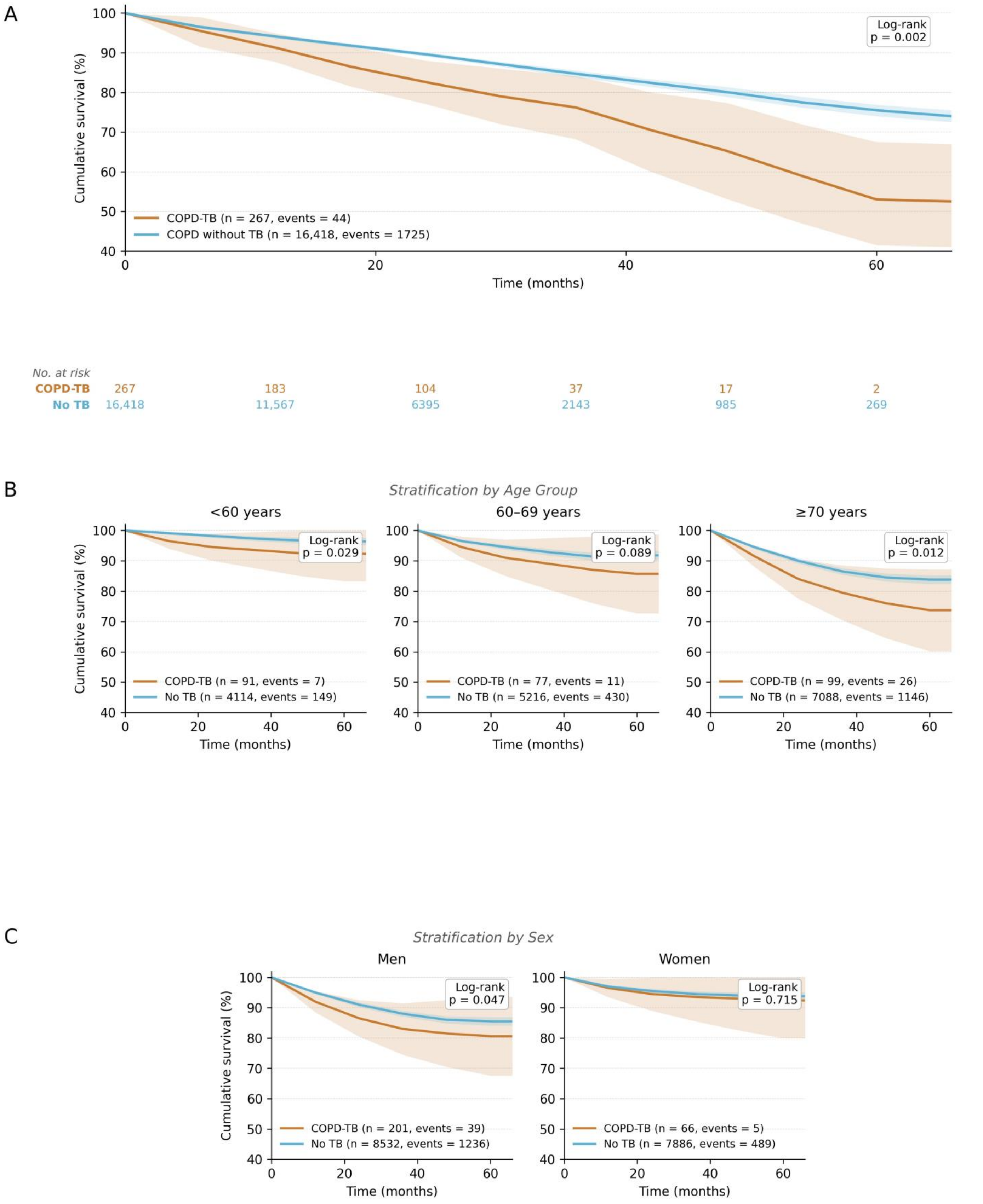

Figure 2. Kaplan–Meier survival curves for patients with COPD according to tuberculosis status (log-rank $p = 0.002$). (A) Whole cohort; (B) stratified by age group (<60, 60–69, and ≥70 years); (C)

stratified by sex (men and women). Shaded bands represent 95% confidence intervals, and the numbers below panel (A) indicate the patients at risk; log-rank *p*-values are shown within each panel.

The highest mortality was observed in patients with post-TB sequelae alone (B90; 19.3%, 38/197), intermediate in those with isolated active TB (A15–A19; 13.6%, 3/22), and lowest in patients coded for both active TB and sequelae (6.2%, 3/48; Table 1). Given the small size of the active-TB-only and combined subgroups, formal statistical comparison between TB manifestations was not performed; these proportions are presented descriptively. These findings suggest that chronic structural damage after TB may have greater long-term prognostic relevance for COPD than the mere presence of recent active infection codes.

3.6. Factors Associated with All-Cause Mortality

To determine whether tuberculosis was associated with mortality risk after adjustment for measured comorbidities, we performed a multivariable analysis (Figure 3).

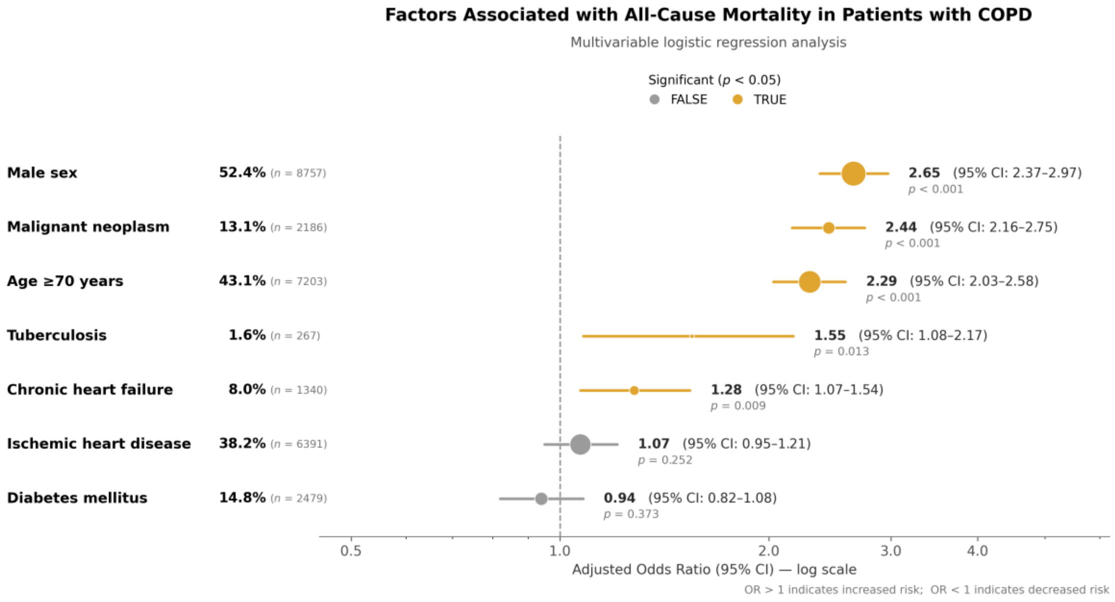

Figure 3. Factors associated with all-cause mortality in patients with COPD: forest plot of adjusted odds ratios from multivariable logistic regression (n = 16,714).

In the multivariable logistic regression model (n = 16,714) adjusted for sex, age, and major comorbidities, tuberculosis remained associated with all-cause mortality after adjustment for available covariates (aOR = 1.55; 95% CI, 1.08–2.17; $p = 0.013$), consistent with the Cox regression estimate (HR = 1.37; 95% CI, 1.01–1.85; $p = 0.040$) (Table 4). The strongest predictors of mortality were male sex (aOR = 2.65; 95% CI, 2.37–2.97; $p < 0.001$), malignant neoplasms (aOR = 2.44; 95% CI, 2.16–2.75; $p < 0.001$), and age ≥ 70 years (aOR = 2.29; 95% CI, 2.03–2.58; $p < 0.001$). Tuberculosis ranked fourth among adverse prognostic factors and was comparable in magnitude to chronic heart failure (aOR = 1.28; 95% CI, 1.08–1.52; $p = 0.005$). In contrast, ischemic heart disease (aOR = 0.96; $p = 0.451$) and diabetes mellitus (aOR = 0.91; $p = 0.213$) were not independently associated with mortality once demographic factors and other comorbidities had been taken into account. The model showed acceptable performance (McFadden pseudo-R^2 = 0.104; AUC = 0.742), values typical for registry-based mortality models that do not include spirometry, smoking status, or pharmacotherapy data.

Table 4. Association of tuberculosis with the main outcomes in patients with COPD (COPD-TB vs. COPD without TB).

Outcome and Model	Effect Estimate (95% CI)	*p*
All-cause mortality		
Unadjusted odds ratio	1.66 (1.20–2.30)	0.003
Adjusted OR (age, sex)	1.61 (1.14–2.28)	0.007
Adjusted OR (multivariable a)	1.55 (1.08–2.17)	0.013
Cox proportional-hazards HR (age, sex)	1.37 (1.01–1.85)	0.040
At least one exacerbation		

Outcome and Model	Effect Estimate (95% CI)	*p*
Unadjusted odds ratio	1.56 (1.14–2.12)	0.006
Adjusted OR (age, sex)	1.43 (1.05–1.96)	0.023
Adjusted OR (age, sex + structural lung disease b)	1.27 (0.93–1.74)	0.136
Frequent exacerbations (≥2)		
Unadjusted odds ratio	1.70 (1.13–2.56)	0.015
Adjusted OR (age, sex)	1.54 (1.02–2.32)	0.041
Adjusted OR (age, sex + structural lung disease b)	1.29 (0.85–1.97)	0.234

Note. Reference: COPD without tuberculosis. Multivariable model adjusted for sex, age, and major comorbidities; structural-lung-disease model additionally adjusted for bronchiectasis, pneumonia, and pulmonary fibrosis. OR, odds ratio; HR, hazard ratio; CI, confidence interval.

In prognostic terms, tuberculosis occupied an intermediate position between the strongest predictors (male sex, malignancy, advanced age) and traditional cardiovascular risk factors. These findings support considering TB history as a nontrivial prognostic marker in routine COPD risk stratification. Because this marker is already present in routine records, it can be incorporated into individualized mortality risk assessment at the point of care without additional testing.

3.7. Profile of Dispensary Follow-Up in Patients with COPD and Tuberculosis

To characterize the medical follow-up of patients with COPD-TB comorbidity, we analyzed codes reflecting specific medical monitoring, diagnostic pathways, and vaccination. TB-specific follow-up codes are summarized in Supplementary Figure S5.

Code Z03.0 (“observation for suspected tuberculosis”) was recorded in 53.2% of patients with COPD-TB, approximately 30 times more often than in patients without TB. This indicates that more than half of the COPD-TB group had undergone TB-specific diagnostic surveillance at some point during follow-up (Supplementary Figure S6).

Code Z03.1 (“observation for suspected malignant neoplasm”) was recorded four times more often in patients with tuberculosis (Supplementary Figure S6), which may reflect the need to differentiate residual post-TB lesions from lung cancer and other focal pulmonary abnormalities.

Patients requiring medical certificates were significantly younger (58.5 vs. 64.7 years; $p = 0.003$) (Supplementary Figure S7), likely reflecting greater social and occupational activity in younger individuals.

The need for medical certificates was highest among patients younger than 70 years (25–27%) and declined sharply in those aged 70 years or older (Supplementary Figure S8).

The overall vaccination frequency in patients with COPD and tuberculosis was significantly lower than in patients without tuberculosis (Supplementary Figure S9). This finding is clinically important because the COPD-TB subgroup is precisely the population in which vaccine-preventable respiratory complications may have the greatest consequences. A summary of the key differences in dispensary follow-up between the groups is presented in Supplementary Table S4. These vaccination findings should be interpreted with caution: ICD-10 Z-codes capture only documented immunization encounters and may not reflect true vaccine uptake or its timing relative to outcomes, so the observed differences may partly reflect differences in coding completeness rather than actual coverage.

Analysis of outpatient follow-up patterns revealed several notable features. More than half of patients with COPD-TB had documentation of TB-specific surveillance, and the need for expert assessment and medical certification was more common in younger age groups. At the same time, vaccination coverage remained suboptimal, identifying a potentially modifiable gap in care.

4. Discussion

This population-based retrospective registry study from the Voronezh Region showed that tuberculosis and its sequelae are clinically meaningful modifiers of COPD course and prognosis. In a cohort of 16,714 patients, tuberculosis or post-TB sequelae were identified in 1.6%, and this subgroup had a distinct clinical profile, higher exacerbation burden, and worse survival.

At the first stage of the analysis, cluster analysis identified five multimorbidity profiles of COPD: cardiovascular, cerebrovascular, hypertensive, polymorbid, and isolated COPD [26]. From a practical standpoint, the most notable finding was the concentration of tuberculosis in the isolated COPD cluster rather than in the polymorbid or cardiovascular profiles. This suggests that TB-associated COPD may reflect, at least in part, a distinct pathway of disease development; however, tuberculosis and COPD share major risk factors—including smoking, lower socioeconomic status and educational attainment, and low body weight—that independently predispose to both conditions, and our registry data cannot disentangle these shared determinants from a direct contribution of tuberculosis [27–29].

The relatively low mortality observed in the polymorbid cluster despite the highest mean number of comorbidities may reflect more intensive follow-up, better treatment adherence, or earlier healthcare contact in heavily monitored patients [30–34]. Because treatment variables were unavailable in the registry, this interpretation remains speculative, but it underscores that multimorbidity count alone does not fully capture prognosis.

The clinical portrait of a patient with COPD-TB in our cohort differed substantially from that of the "typical" patient with COPD. These patients were younger, predominantly male, and less likely to have obesity, diabetes, or hypertension, yet they were far more likely to have bronchiectasis, fibrosis, and pneumonia. This pattern supports the concept of a structurally damaged, infection-prone COPD phenotype linked to prior TB [24,35,36]. Several factors may contribute to this pattern. Patients with COPD-TB were younger, and because obesity, diabetes, and hypertension become more prevalent with age, the age difference partly explains their lower cardiometabolic burden. Tuberculosis is also associated with low body weight and a catabolic, frequently undernourished phenotype, which is inversely related to obesity and type 2 diabetes. Differential health-seeking and coding—with clinical attention concentrated on respiratory and infectious problems in this subgroup—together with selection effects inherent to registry data, may further reduce the recorded prevalence of metabolic conditions. These explanations are not mutually exclusive and remain hypotheses that cannot be confirmed with the available data.

Most patients in the present cohort had post-tuberculosis lung disease rather than active TB. This distinction is clinically important because PTLD often remains underrecognized after completion of anti-TB treatment, despite persistent airflow limitation, radiographic abnormalities, and impaired quality of life [37,38]. Our findings suggest that PTLD should be viewed not as a resolved historical condition but as an active determinant of COPD prognosis.

The presence of tuberculosis or its sequelae was associated with a significant increase in COPD exacerbations (Supplementary Figure S3). Patients with TB history were more likely to experience at least one exacerbation, had a higher mean number of exacerbations,

and showed a tendency toward the frequent-exacerbator phenotype. These associations remained clinically relevant after adjustment, indicating that TB is not merely a marker of disease complexity but may contribute directly to instability of the disease course [39].

Seasonal analysis demonstrated a spring shift in exacerbation peak among patients with COPD and TB history. This may indicate a trigger pattern different from that of classic COPD, possibly mediated by residual structural damage, altered local immunity, and susceptibility to respiratory infections after winter [40–43]. This finding requires confirmation in other cohorts and should be regarded as hypothesis-generating rather than clinically actionable.

Our results also demonstrated an adverse effect of tuberculosis and its sequelae on long-term COPD prognosis [13,44–47]. Mortality was higher in the COPD-TB group, and both Cox regression and multivariable logistic regression confirmed an independent association between TB and death. In practical terms, this means that TB history should be incorporated into risk stratification when planning long-term follow-up of patients with COPD.

A noteworthy finding was the heterogeneity of mortality across TB manifestations. Mortality was highest among patients with post-TB sequelae rather than among those with active TB [48,49]. This may indicate that chronic structural damage and long-term loss of pulmonary reserve have greater prognostic importance than recent infection status alone [50–52]. It also suggests that reliance solely on active TB coding may underestimate the true burden of TB-related respiratory vulnerability.

The study period overlapped with the COVID-19 pandemic, which may have influenced both TB detection [53] and COPD outcomes [54]. Nevertheless, the observed associations were robust and clinically coherent. Analysis of coded comorbidities also showed that the COPD-TB subgroup had lower cardiometabolic burden but worse respiratory structural disease, reinforcing the internal consistency of the phenotype identified.

The most practice-oriented conclusion of this study is that patients with COPD and a history of tuberculosis should be recognized as a high-risk subgroup. These patients may benefit from intensified follow-up, active prevention of exacerbations, structured vaccination strategies, and, where available, more detailed functional and radiologic assessment to identify PTLD-related damage.

These observations have direct implications for the personalized management of COPD. Because a history of tuberculosis and post-tuberculosis lung changes are documented in routine records, they can serve as readily accessible markers for individualizing care. Patients with COPD-TB may warrant a higher intensity of follow-up and shorter intervals between scheduled visits, with proactive rather than deferred exacerbation-prevention measures, including timely counseling on influenza and pneumococcal vaccination. Their distinctive, structurally damaged and infection-prone profile also argues for closer functional and radiological monitoring and for clearer referral pathways between pulmonology and tuberculosis services. Embedding TB-related lung history into individual risk stratification would help shift COPD care from a uniform model toward a personalized strategy in which surveillance and prevention are matched to each patient's risk.

Study Limitations

The findings should be interpreted in light of the limitations inherent to a retrospective registry-based study using ICD-10 codes. Misclassification of diagnoses and outcomes is possible; microbiological verification was unavailable; and residual confounding cannot be excluded. The database also did not allow differentiation between incident and long-standing COPD in all cases or provide standardized radiologic characterization of post-TB changes. Because each dated J44.0/J44.1 record was counted as an exacerbation episode, repeated coding within a single care episode may have overestimated

exacerbation counts; this potential misclassification is expected to be non-differential with respect to tuberculosis status. Several further limitations should be emphasized. COPD diagnoses were established by treating physicians in accordance with current clinical guidelines, which require spirometric confirmation of airflow limitation; however, the underlying spirometry values (FEV1 and FEV1/FVC) were not available in the de-identified dataset and could not be used for severity grading or sensitivity analyses. Important confounders were unavailable, including smoking history and intensity, biomass exposure, body mass index, pulmonary-function parameters (FEV1 and FVC, which are especially relevant in the TB group), pharmacological treatment, pulmonary rehabilitation, and socioeconomic status. Because smoking and low body weight are strongly associated with both tuberculosis and COPD outcomes, residual confounding is likely, and causal interpretation should remain cautious; accordingly, associations are reported as adjusted for available covariates rather than as proof of independent causation. The COPD-TB subgroup was relatively small (n = 267), limiting statistical power and the stability of subgroup estimates, and the median follow-up was short for two chronic, progressive diseases, so longer-term differences may be underestimated. Finally, combining active tuberculosis and post-tuberculosis sequelae into a single category increased statistical power but is clinically heterogeneous; the prognostic signal was driven mainly by post-tuberculosis lung disease, and active and chronic post-TB disease should not be assumed to carry equivalent prognostic weight.

5. Conclusions

Tuberculosis, and especially post-tuberculosis lung disease, is associated with a distinct COPD phenotype characterized by younger age, male predominance, lower cardiometabolic burden, more frequent bronchiectasis and fibrosis, higher exacerbation burden, and worse survival. Tuberculosis remained associated with mortality after adjustment for available covariates, whereas its association with COPD exacerbations was largely mediated by post-tuberculosis structural lung disease. These findings support inclusion of TB history in COPD risk stratification and highlight the need for closer longitudinal follow-up of patients with COPD-TB. More broadly, a TB-related lung history should be considered a clinically accessible marker for risk stratification in personalized COPD management: because it is readily available from routine records and identifies patients with a distinct prognosis, it can be used to individualize follow-up intensity, monitoring, and exacerbation-prevention strategies.

Supplementary Materials: The following supporting information can be downloaded at: https://www.mdpi.com/article/doi/s1, Figure S1: Age and sex distribution of patients with COPD; Figure S2: Incidence rate of active tuberculosis (A15–A19) among patients with COPD by year of observation; Figure S3: Distribution of patients by number of COPD exacerbations according to tuberculosis status; Figure S4: Monthly distribution of COPD exacerbations in patients with and without tuberculosis; Figure S5: Frequency of tuberculosis-specific follow-up codes (Z03.0) according to tuberculosis status; Figure S6: Frequency of diagnostic observation codes (Z03.1, Z03.8) according to tuberculosis status; Figure S7: Demographic and clinical characteristics of patients requiring a medical certificate (Z02.7); Figure S8: Need for medical certificates (Z02.7) by age group; Figure S9: Vaccination coverage according to tuberculosis status; Table S1: Frequency of comorbid conditions in the overall COPD cohort; Table S2: Multimorbidity profiles identified by K-means clustering, and the distribution of tuberculosis; Table S3: Vaccination frequency according to tuberculosis status; Table S4: Summary profile of dispensary follow-up according to tuberculosis status; Table S5: Stratified analysis of all-cause mortality according to tuberculosis status.

Author Contributions: Conceptualization, S.K.; methodology, S.K.; validation, D.O.; formal analysis, S.K.; resources, S.K. and D.O.; data curation, S.K. and D.O.; writing—original draft preparation, D.O.; writing—review and editing, S.K. and D.O.; visualization, S.K. and D.O.; supervision, S.K.; project administration, S.K. All authors have read and agreed to the published version of the manuscript.

Funding: This research received no external funding.

Institutional Review Board Statement: The study was approved by the Local Ethics Committee of Ryazan State Medical University, Russian Ministry of Health (Protocol No. 4, 6 December 2024). The Ethics Committee approved the use of data from 2019 to 2025 obtained from the regional health information system.

Informed Consent Statement: The study used de-identified administrative healthcare data; therefore, the requirement for informed consent was waived by the Local Ethics Committee. The records were anonymized by the data provider before being shared with the research team so that no identifiable personal information was available to the investigators.

Data Availability Statement: The de-identified data are available from the corresponding author upon reasonable request.

Conflicts of Interest: The authors declare no conflicts of interest.

References

1. Safiri, S.; Carson-Chahhoud, K.; Noori, M.; Nejadghaderi, S.A.; Sullman, M.J.M.; Ahmadian Heris, J.; Ansarin, K.; Mansournia, M.A.; Collins, G.S.; Kolahi, A.-A.; et al. Burden of Chronic Obstructive Pulmonary Disease and Its Attributable Risk Factors in 204 Countries and Territories, 1990–2019: Results from the Global Burden of Disease Study 2019. *BMJ* **2022**, *378*, e069679. https://doi.org/10.1136/bmj-2021-069679.
2. Zhamakurova, A.N.; Kuanyshkalieva, A.S.; Smailova, D.S.; Kulzhanov, M.K. Analiz zabolevaemosti khronicheskoy obstruktivnoy bolezni legkikh v Respublike Kazakhstan [Analysis of the Incidence of Chronic Obstructive Pulmonary Disease in the Republic of Kazakhstan]. *Farm. Kaz.* **2024**, *2*, 49–56. https://doi.org/10.53511/pharmkaz.2024.60.66.007.
3. Adeloye, D.; Song, P.; Zhu, Y.; Campbell, H.; Sheikh, A.; Rudan, I. Global, Regional, and National Prevalence of, and Risk Factors for, Chronic Obstructive Pulmonary Disease (COPD) in 2019: A Systematic Review and Modelling Analysis. *Lancet Respir. Med.* **2022**, *10*, 447–458. https://doi.org/10.1016/S2213-2600(21)00511-7.
4. Berry, C.E.; Wise, R.A. Mortality in COPD: Causes, Risk Factors, and Prevention. *COPD J. Chronic Obstr. Pulm. Dis.* **2010**, *7*, 375–382. https://doi.org/10.3109/15412555.2010.510160.
5. Sidhaye, V.K.; Nishida, K.; Martinez, F.J. Precision Medicine in COPD: Where Are We and Where Do We Need to Go? *Eur. Respir. Rev.* **2018**, *27*, 180022. https://doi.org/10.1183/16000617.0022-2018.
6. Agusti, A.; Faner, R. Personalizing COPD Care: Phenotypes, Endotypes, GETomics, the Trajectome, Syndemics and Treatable Traits. *La Presse Médicale* **2026**, *55*, 104318. https://doi.org/10.1016/j.lpm.2025.104318.
7. Alupo, P.; Mugenyi, L.; Katagira, W.; Kayongo, A.; Nalunjogi, J.; Siddharthan, T.; Hurst, J.R.; Kirenga, B.; Jones, R. Characteristics and Phenotypes of a COPD Cohort from Referral Hospital Clinics in Uganda. *BMJ Open Respir. Res.* **2024**, *11*, e001816. https://doi.org/10.1136/bmjresp-2023-001816.
8. Cioboata, R.; Vlasceanu, S.G.; Mitroi, D.M.; Zlatian, O.M.; Balteanu, M.A.; Andrei, G.M.; Biciusca, V.; Olteanu, M. History of Pulmonary Tuberculosis Accelerates Early Onset and Severity of COPD: Evidence from a Multicenter Study in Romania. *J. Clin. Med.* **2025**, *14*, 5980. https://doi.org/10.3390/jcm14175980.
9. Tobin, E.H.; Tristram, D. Tuberculosis Overview. In *StatPearls*; StatPearls Publishing: Treasure Island, FL, USA, 2025.
10. Joo, D.-H.; Kim, M.C.; Sin, S.; Kang, H.-R.; Song, J.H.; Kim, H.-J.; Song, M.J.; Kwon, B.S.; Kim, Y.W.; Lee, Y.J.; et al. Incidence and Risk Factors of Tuberculosis-Associated Chronic Obstructive Pulmonary Disease. *Int. J. Chron. Obs. Pulmon Dis.* **2025**, *20*, 2091–2102. https://doi.org/10.2147/COPD.S523732.
11. Gai, X.; Allwood, B.; Sun, Y. Advances in the Awareness of Tuberculosis-Associated Chronic Obstructive Pulmonary Disease. *Chin. Med. J. Pulm. Crit. Care Med.* **2024**, *2*, 250–256. https://doi.org/10.1016/j.pccm.2024.08.008.
12. Zavala, M.J.; Becker, G.L.; Blount, R.J. Interrelationships between Tuberculosis and Chronic Obstructive Pulmonary Disease. *Curr. Opin. Pulm. Med.* **2023**, *29*, 104–111. https://doi.org/10.1097/MCP.0000000000000938.

13. Jin, J.; Yu, W.; Li, S.; Lu, L.; Liu, X.; Sun, Y. Factors Associated with Bronchiectasis in Patients with Moderate–Severe Chronic Obstructive Pulmonary Disease. *Medicine* **2016**, *95*, e4219. https://doi.org/10.1097/MD.0000000000004219.
14. Amaral, A.F.S.; Coton, S.; Kato, B.; Tan, W.C.; Studnicka, M.; Janson, C.; Gislason, T.; Mannino, D.; Bateman, E.D.; Buist, S.; et al. Tuberculosis Associates with Both Airflow Obstruction and Low Lung Function: BOLD Results. *Eur. Respir. J.* **2015**, *46*, 1104–1112. https://doi.org/10.1183/13993003.02325-2014.
15. Thu, W.P.P.; Hu, T.H.; D'Ambrosio, L.; Centis, R.; Ong, C.W.M.; Migliori, G.B. Charting the Course in Post-Tuberculosis Lung Disease: From Inflammation to Intervention. *Arch. Bronconeumol.* **2025**, *61*, 757–765. https://doi.org/10.1016/j.arbres.2025.09.017.
16. Fan, H.; Wu, F.; Liu, J.; Zeng, W.; Zheng, S.; Tian, H.; Li, H.; Yang, H.; Wang, Z.; Deng, Z.; et al. Pulmonary Tuberculosis as a Risk Factor for Chronic Obstructive Pulmonary Disease: A Systematic Review and Meta-Analysis. *Ann. Transl. Med.* **2021**, *9*, 390. https://doi.org/10.21037/atm-20-4576.
17. Kamenar, K.; Hossen, S.; Gupte, A.N.; Siddharthan, T.; Pollard, S.; Chowdhury, M.; Rubinstein, A.L.; Irazola, V.E.; Gutierrez, L.; Miranda, J.J.; et al. Previous Tuberculosis Disease as a Risk Factor for Chronic Obstructive Pulmonary Disease: A Cross-Sectional Analysis of Multicountry, Population-Based Studies. *Thorax* **2022**, *77*, 1088–1097. https://doi.org/10.1136/thoraxjnl-2020-216500.
18. Feng, J.; Hu, M.; Duan, H. Bidirectional Association Between Tuberculosis and Chronic Obstructive Pulmonary Disease: A Systematic Review and Meta-Analysis. *J. Clin. Med.* **2025**, *14*, 7639. https://doi.org/10.3390/jcm14217639.
19. Sy, K.T.L.; Horváth-Puhó, E.; Sørensen, H.T.; Szépligeti, S.K.; Heeren, T.C.; Thomsen, R.W.; Fox, M.P.; Horsburgh, Jr., C.R. Burden of Chronic Obstructive Pulmonary Disease Attributable to Tuberculosis: A Microsimulation Study. *Am. J. Epidemiol.* **2023**, *192*, 908–915. https://doi.org/10.1093/aje/kwad042.
20. Rhee, C.K.; Yoo, K.H.; Lee, J.H.; Park, M.J.; Kim, W.J.; Park, Y.B.; Hwang, Y.I.; Kim, Y.S.; Jung, J.Y.; Moon, J.Y.; et al. Clinical Characteristics of Patients with Tuberculosis-Destroyed Lung. *Int. J. Tuberc. Lung Dis.* **2013**, *17*, 67–75. https://doi.org/10.5588/ijtld.12.0351.
21. Sarkar, M.; Srinivasa; Madabhavi, I.; Kumar, K. Tuberculosis Associated Chronic Obstructive Pulmonary Disease. *Clin. Respir. J.* **2017**, *11*, 285–295. https://doi.org/10.1111/crj.12621.
22. Allwood, B.W.; Maasdorp, E.; Kim, G.J.; Cooper, C.; Goldin, J.; Van Zyl-Smit, R.N.; Bateman, E.D.; Dawson, R. Transition from Restrictive to Obstructive Lung Function Impairment During Treatment and Follow-Up of Active Tuberculosis. *Int. J. Chronic Obstr. Pulm. Dis.* **2020**, *15*, 1039–1047. https://doi.org/10.2147/COPD.S219731.
23. Zeng, Z.; Chen, H.; Shao, Z.; Guan, Y.; Zhan, Y.; Cao, X.; Li, S.; Hao, X. Associations of Prior Pulmonary Tuberculosis with the Incident COPD: A Prospective Cohort Study. *Ther. Adv. Respir. Dis.* **2024**, *18*, 17534666241239455. https://doi.org/10.1177/17534666241239455.
24. Venkatesan, P. GOLD COPD Report: 2025 Update. *Lancet Respir. Med.* **2025**, *13*, e7–e8. https://doi.org/10.1016/S2213-2600(24)00413-2.
25. Kotlyarov, S.; Lyubavin, A. Structure of Comorbidities and Causes of Death in Patients with Atrial Fibrillation and Chronic Obstructive Pulmonary Disease. *J. Clin. Med.* **2025**, *14*, 5045. https://doi.org/10.3390/jcm14145045.
26. Chen, H.; Chen, D.; Zhang, J.; Chen, Y. Cluster Analysis of Comorbidity Profiles Reveals Heterogeneity in Hospitalized Patients with Chronic Obstructive Pulmonary Disease. *Chin. Med. J.* **2025**, *138*, 475–477. https://doi.org/10.1097/CM9.0000000000003370.
27. Mariniello, D.F.; D'Agnano, V.; Cennamo, D.; Conte, S.; Quarcio, G.; Notizia, L.; Pagliaro, R.; Schiattarella, A.; Salvi, R.; Bianco, A.; et al. Comorbidities in COPD: Current and Future Treatment Challenges. *J. Clin. Med.* **2024**, *13*, 743. https://doi.org/10.3390/jcm13030743.
28. Chien, S.-Y.; Hu, H.-C.; Cho, H.-Y. Long-Term Monitoring of Individuals with Chronic Obstructive Pulmonary Disease Using Digital Health Technology: Qualitative Study. *J. Med. Internet Res.* **2025**, *27*, e63660. https://doi.org/10.2196/63660.
29. Allwood, B.W.; Byrne, A.; Meghji, J.; Rachow, A.; Van Der Zalm, M.M.; Schoch, O.D. Post-Tuberculosis Lung Disease: Clinical Review of an Under-Recognised Global Challenge. *Respiration* **2021**, *100*, 751–763. https://doi.org/10.1159/000512531.
30. Roche, N.; Molinari, N.; Watier, L.; Schmidt, A.; Panes, A.; Pagès, N.; Perrier, S.; Vataire, A.-L.; Marcadé-Fulcrand, V.; Bourdin, A. Characteristics and Real-World Health Clinical Outcomes of Uncontrolled COPD Patients: Population-Based Study in France. *ERJ Open Res.* **2025**, *11*, 00104–02025. https://doi.org/10.1183/23120541.00104-2025.
31. Bagisheva, N.V.; Mordyk, A.V.; Viktorova, I.A.; Trukhan, D.I. Cardiovascular Pathology in Patients with Newly Diagnosed Tuberculosis and Chronic Obstructive Pulmonary Disease. *Med. Sov.* **2021**, *14*, 142–148. https://doi.org/10.21518/2079-701X-2021-14-142-148.
32. Uryasyev, O.M.; Panfilov, Y.A.; Granatkin, M.A.; Pyko, A.A. CHRONIC OBSTRUCTIVE PULMONARY DISEASE AND COMORBIDITY. *J. Volgogr. State Med. Univ.* **2020**, *17*, 174–177. https://doi.org/10.19163/1994-9480-2020-2(74)-174-177.

33. Chaulin, A.M.; Duplyakov, D.V. Comorbidity in Chronic Obstructive Pulmonary Disease and Cardiovascular Disease. *Cardiovasc. Ther. Prev.* **2021**, *20*, 2539. https://doi.org/10.15829/1728-8800-2021-2539.
34. Bagisheva, N.V.; Mordyk, A.V.; Moiseeva, M.V.; Nesterova, K.I.; Nebesnaya, E.Y.; Filipenko, G.V.; Aroyan, A.R.; Batishcheva, T.L.; Andreeva, I.A. Vozmozhnosti kardioprotektivnoy terapii: V fokuse patsienty s tuberkulezom i khronicheskoy obstruktivnoy bolezn'yu legkikh [Possibilities of Cardioprotective Therapy: Focus on Patients with Tuberculosis and Chronic Obstructive Pulmonary Disease]. *Вестник ЦНИИТ* **2021**, *1*, 65–75. https://doi.org/10.7868/S2587667821010088.
35. Sehgal, I.S.; Dhooria, S.; Muthu, V.; Salzer, H.J.F.; Agarwal, R. Burden, Clinical Features, and Outcomes of Post-Tuberculosis Chronic Obstructive Lung Diseases. *Curr. Opin. Pulm. Med.* **2024**, *30*, 156–166. https://doi.org/10.1097/MCP.0000000000001026.
36. Solarat, B.; Perea, L.; Faner, R.; De La Rosa, D.; Martínez-García, M.Á.; Sibila, O. Pathophysiology of Chronic Bronchial Infection in Bronchiectasis. *Arch. Bronconeumol.* **2023**, *59*, 101–108. https://doi.org/10.1016/j.arbres.2022.09.004.
37. Byrne, A.L.; Marais, B.J.; Mitnick, C.D.; Lecca, L.; Marks, G.B. Tuberculosis and Chronic Respiratory Disease: A Systematic Review. *Int. J. Infect. Dis.* **2015**, *32*, 138–146. https://doi.org/10.1016/j.ijid.2014.12.016.
38. Ivanova, O.; Hoffmann, V.S.; Lange, C.; Hoelscher, M.; Rachow, A. Post-Tuberculosis Lung Impairment: Systematic Review and Meta-Analysis of Spirometry Data from 14 621 People. *Eur. Respir. Rev.* **2023**, *32*, 220221. https://doi.org/10.1183/16000617.0221-2022.
39. Oh, J.Y.; Lee, Y.S.; Min, K.H.; Hur, G.Y.; Lee, S.Y.; Kang, K.H.; Rhee, C.K.; Park, S.J.; Shim, J.J. Difference in Systemic Inflammation and Predictors of Acute Exacerbation between Smoking-Associated COPD and Tuberculosis-Associated COPD. *Int. J. Chronic Obstr. Pulm. Dis.* **2018**, *13*, 3381–3387. https://doi.org/10.2147/COPD.S177371.
40. Meghji, J.; Lesosky, M.; Joekes, E.; Banda, P.; Rylance, J.; Gordon, S.; Jacob, J.; Zonderland, H.; MacPherson, P.; Corbett, E.L.; et al. Patient Outcomes Associated with Post-Tuberculosis Lung Damage in Malawi: A Prospective Cohort Study. *Thorax* **2020**, *75*, 269–278. https://doi.org/10.1136/thoraxjnl-2019-213808.
41. Charles, T.; Eckardt, M.; Karo, B.; Haas, W.; Kröger, S. Seasonality in Extra-Pulmonary Tuberculosis Notifications in Germany 2004–2014- a Time Series Analysis. *BMC Public Health* **2021**, *21*, 661. https://doi.org/10.1186/s12889-021-10655-6.
42. Martineau, A.R.; Nhamoyebonde, S.; Oni, T.; Rangaka, M.X.; Marais, S.; Bangani, N.; Tsekela, R.; Bashe, L.; De Azevedo, V.; Caldwell, J.; et al. Reciprocal Seasonal Variation in Vitamin D Status and Tuberculosis Notifications in Cape Town, South Africa. *Proc. Natl. Acad. Sci. USA* **2011**, *108*, 19013–19017. https://doi.org/10.1073/pnas.1111825108.
43. Tedijanto, C.; Hermans, S.; Cobelens, F.; Wood, R.; Andrews, J.R. Drivers of Seasonal Variation in Tuberculosis Incidence: Insights from a Systematic Review and Mathematical Model. *Epidemiology* **2018**, *29*, 857–866. https://doi.org/10.1097/EDE.0000000000000877.
44. Yakar, H.I.; Gunen, H.; Pehlivan, E.; Aydogan, S. The Role of Tuberculosis in COPD. *Int. J. Chronic Obstr. Pulm. Dis.* **2017**, *12*, 323–329. https://doi.org/10.2147/COPD.S116086.
45. Wang, J.; Yuan, B.; Fang, Y.; Zhao, C.; Liu, J.; Xiong, K. Post-Tuberculosis Morbidities and Their Associated Mortality: Moving from Challenges to Solutions. *Eur. Respir. Rev.* **2025**, *34*, 250148. https://doi.org/10.1183/16000617.0148-2025.
46. Choi, H.; Han, K.; Jung, J.-H.; Park, S.H.; Kim, S.H.; Kang, H.K.; Sohn, J.W.; Shin, D.W.; Lee, H. Long-Term Mortality of Tuberculosis Survivors in Korea: A Population-Based Longitudinal Study. *Clin. Infect. Dis.* **2023**, *76*, e973–e981. https://doi.org/10.1093/cid/ciac411.
47. Romanowski, K.; Baumann, B.; Basham, C.A.; Ahmad Khan, F.; Fox, G.J.; Johnston, J.C. Long-Term All-Cause Mortality in People Treated for Tuberculosis: A Systematic Review and Meta-Analysis. *Lancet Infect. Dis.* **2019**, *19*, 1129–1137. https://doi.org/10.1016/S1473-3099(19)30309-3.
48. WHO. *WHO Operational Handbook on Tuberculosis. Module 4: Treatment—Tuberculosis Care and Support*, 1st ed.; World Health Organization: Geneva, Switzerland, 2022.
49. Migliori, G.B.; Marx, F.M.; Ambrosino, N.; Zampogna, E.; Schaaf, H.S.; Van Der Zalm, M.M.; Allwood, B.; Byrne, A.L.; Mortimer, K.; Wallis, R.S.; et al. Clinical Standards for the Assessment, Management and Rehabilitation of Post-TB Lung Disease. *Int. J. Tuberc. Lung Dis.* **2021**, *25*, 797–813. https://doi.org/10.5588/ijtld.21.0425.
50. Meghji, J.; Auld, S.C.; Bisson, G.P.; Khosa, C.; Masekela, R.; Navuluri, N.; Rachow, A. Post-Tuberculosis Lung Disease: Towards Prevention, Diagnosis, and Care. *Lancet Respir. Med.* **2025**, *13*, 460–472. https://doi.org/10.1016/S2213-2600(24)00429-6.
51. Tayutina, T.V.; Bortnikov, A.Y.; Lysenko, A.V.; Kazarya, T.M.; Eknadiosova, D.V. The Structure of Mortality and Concomitant Pathology in Patients with Chronic Obstructive Pulmonary Disease: Parallels with Pathomorphological Pulmonary Changes. *Med. Theory Pract.* **2024**, *9*, 4–17. https://doi.org/10.56871/MTP.2024.61.67.001.

52. Menzies, N.A.; Marks, S.M.; Hsieh, Y.L.; Swartwood, N.A.; Beeler Asay, G.R.; Skarbinski, J.; Horsburgh, C.R.; Cohen, T. Contribution of Posttuberculosis Sequelae to Life-Years Lost from Tuberculosis Disease in the United States, 2015–2019. *Am. J. Respir. Crit. Care Med.* **2025**, *211*, 1059–1068. https://doi.org/10.1164/rccm.202411-2213OC.
53. WHO. *Global Tuberculosis Report 2022*, 1st ed.; World Health Organization: Geneva, Switzerland, 2022.
54. Choi, J.Y.; Kim, K.J.; Rhee, C.K. Change in Exacerbation Rate of COPD Patients before and after COVID-19 Infection. *Sci. Rep.* **2025**, *15*, 2427. https://doi.org/10.1038/s41598-025-86426-9.

Disclaimer/Publisher's Note: The statements, opinions and data contained in all publications are solely those of the individual author(s) and contributor(s) and not of MDPI and/or the editor(s). MDPI and/or the editor(s) disclaim responsibility for any injury to people or property resulting from any ideas, methods, instructions or products referred to in the content.
